# Intimate partner violence as seen in post-conflict eastern Uganda: prevalence, risk factors and mental health consequences

**DOI:** 10.1186/s12914-016-0079-x

**Published:** 2016-01-29

**Authors:** Eugene Kinyanda, Helen A Weiss, Margaret Mungherera, Patrick Onyango-Mangen, Emmanuel Ngabirano, Rehema Kajungu, Johnson Kagugube, Wilson Muhwezi, Julius Muron, Vikram Patel

**Affiliations:** 1 MRC/UVRI Uganda Research Unit on AIDS/MRC/DFID African Leadership Award, P.O. Box 49, Entebbe, Uganda; 2 London School of Hygiene and Tropical Medicine, London, WC1E 7HT UK; 3 Mulago National Referral Hospital, P.O. Box 7051, Kampala, Uganda; 4 Transcultural Psychosocial Organisation, P.O. Box 21646, Kampala, Uganda; 5 Uganda Bureau of Statistics, P.O. Box 7186, Kampala, Uganda; 6 Department of Psychiatry, Makerere University, P.O. Box 7072, Kampala, Uganda; 7 Butabika National Psychiatric Referral Hospital, P.O. Box 7017, Kampala, Uganda; 8 London School of Hygiene & Tropical Medicine and Sangath, Sangath Centre, 841/1 Alto Porvorim, Goa, 403521 India

**Keywords:** Intimate partner violence, Post-conflict, Africa, Risk factors, Mental health consequences

## Abstract

**Background:**

Conflict and post-conflict communities in sub-Saharan Africa have a high under recognised problem of intimate partner violence (IPV). Part of the reason for this has been the limited data on IPV from conflict affected sub-Saharan Africa. This paper reports on the prevalence, risk factors and mental health consequences of IPV victimisation in both gender as seen in post-conflict eastern Uganda.

**Methods:**

A cross-sectional survey was carried out in two districts of eastern Uganda. The primary outcome of IPV victimisation was assessed using a modified Intimate Partner Violence assessment questionnaire of the American Congress of Obstetricians and Gynaecologists.

**Results:**

The prevalence of any form of IPV victimisation (physical and/or sexual and/or psychological IPV) in this study was 43.7 % [95 % CI, 40.1–47.4 %], with no statistically significant difference between the two gender. The factors significantly associated with IPV victimisation were: sub-county (representing ecological factors), poverty, use of alcohol, and physical and sexual war torture experiences. The mental health problems associated with IPV victimisation were probable problem alcohol drinking, attempted suicide and probable major depressive disorder.

**Conclusion:**

In post-conflict eastern Uganda, in both gender, war torture was a risk factor for IPV victimisation and IPV victimisation was associated with mental health problems.

## Background

Intimate partner violence (IPV) in conflict and post-conflict situation has not received as much attention as conflict related violence, the latter having been the subject of numerous UN Security Council resolutions in the last decade [1]. Part of the reason for this has been the limited evidence on IPV in conflict and post-conflict situations. Stark and Ager (2011) in systematic review involving 10 studies on the prevalence of gender-based violence in complex emergencies reported that the rates of IPV were much higher than rates of wartime rape and sexual violence perpetrated by individuals outside the home [2].

Looking at the limited evidence on IPV from conflict and post-conflict sub-Saharan African countries points towards a serious public health problem. Data from recent Demographic and Health Surveys (DHS) and other cross-sectional studies undertaken on IPV victimisation among partnered or ever partnered females reveals the following picture: Democratic Republic of Congo (prevalence for any form of IPV of 57 % ) [3]; Ivory Coast (prevalence of 49.8 % for lifetime physical and/or sexual IPV) [4]; South Sudan (prevalence for physical IPV of 20 % -only estimates available) [5]; Liberia (prevalence of 37.9 % for lifetime any form of IPV) [6]; and Uganda (prevalence of 59.7 % for any form of IPV (physical and/or sexual and/or psychological IPV [7]. Despite even more limited data, the available evidence on male IPV victimisation in conflict and post-conflict settings in sub-Saharan Africa suggests that it is a significant public health problem, with the following rates reported: Democratic Republic of Congo (prevalence of 18.8 % for ever experienced physical IPV) [8]; Uganda (prevalence of 42.3 % for any form of IPV (physical and/or sexual and/or psychological IPV [7]. Liberia (prevalence of 31.3 % for lifetime any form of IPV) [6]; and Ivory Coast (prevalence of 19.8 % for lifetime physical IPV) [4].

While there is an established body of literature on the mental health consequences of IPV victimisation in non-conflict situations, there is a paucity of data on the relationship between conflict-related violence, IPV victimisation and mental disorders including a gender differentiated analysis. Among the few studies that have been undertaken in this area, Usta and colleagues (2008) in Lebanon observed that women's self-reported negative mental health scores were positively correlated with both conflict-related violence and IPV during and after the conflict [9]. Gupta and colleagues (2014) in a war affected population in rural Ivory Coast reported a positive robust relationship between past-year IPV and current probable PTSD [10]. Kinyanda and colleagues (2009) in a cross-sectional study in post-conflict Liberia observed that physical torture, psychological torture and IPV victimisation were each significantly associated with psychological distress in both gender, while sexual torture was significantly associated with psychological distress only among males [6].

We undertook this study in rural post-conflict rural eastern Uganda to investigate the prevalence of IPV victimisation, war torture risk factors and possible mental health consequences including undertaking a gender differentiated analysis of these factors. We hypothesised that in post-conflict eastern Uganda, war torture is a risk factor of IPV victimisation in both gender and that mental health problems are a consequence of IPV victimisation.

This study was undertaken in Uganda a country which has suffered chronic war conflict for most of its post-independence history. It is only until 2006 that the 20 year old conflict in northern and eastern Uganda between the Lord’s Resistance Army (LRA) and government ended and even more recently in 2010 that the government successfully stopped armed cattle rustling that had devastated north-eastern Uganda [11, 12].

The Teso sub-region (approximate population 3.2 million people) where this study was undertaken is found in Eastern Uganda. The people of this region belong to the cattle keeping Nilo-hamite ethnic group. This region has suffered centuries of conflict and human rights abuses which were initially confined to low scale cattle rustling raids by the neighbouring tribes who were initially only armed with spears. The violent politics that characterised Uganda’s post-independence history introduced new dimensions and elements to this conflict including: the fall of the Idi Amin’s regime in 1979 introduced small arms to the cattle rustling raids and commercialised the cattle raids; between 1986–1992, there was a locally based armed rebellion by Ugandan People’s Army (UPA) against the central government; and in 2003 the sub-region suffered incursions by the vicious Lord’s Resistance Army (LRA) who were based in northern Uganda [13, 14]. As a result of all this conflict the sub-region has suffered gross human rights abuses including killings, mutilations, abductions, torture, displacement into IDP camps, loss of property and the complete destruction of the sub-region’s herd of cattle and with it a centuries old way of life that revolved around the cattle [13, 14]. The data for this study was collected between September 2008 and October 2009 just as the sub-region was emerging from this conflict.

## Methods

A cross-sectional survey was carried out in four sub-counties in the two districts of Amuria and Katakwi in war affected Eastern Uganda. This study was nested within a Uganda AIDS Commission (Civil Society Fund) funded project that was implemented to address HIV related psychiatric and psychosocial vulnerabilities in the war affected community. This study was carried out in parallel to the other activities of the Civil Society Funded project and has so far led to two publications [15, 16].

### Sampling procedure

The inclusion criteria for this study was: must have been a resident of the 4 study sub-counties; an adult (18 years and above) or a mature minor (14–17 years as defined by the national guidelines) [17]; must be conversant with the Itesot language (language in which the questionnaire was translated); able to understand the study questionnaire. The exclusion criteria was being too sick or cognitive impaired not to understand the study questionnaire.

A multistage sampling procedure was used where a representative sample of both vulnerable and non-vulnerable individuals was recruited into the study. A vulnerable individual for purposes of this study was defined as having any of the following characteristics: widowed, divorced or separated; living in an internally displaced persons camp; women who had suffered sexual torture; single mothers; orphans; out of school youth; child/adolescent mothers; very poor women and adolescent girls; having a mental illness; survivor of intimate partner violence; and survivors of recent famines and floods, derived from previous studies undertaken by the research team among war affected populations [11, 13, 18]. Non-vulnerable individuals were those who did not have any of the above characteristics. This sample was drawn from the Teso sub-region from four sub-counties in the districts of Amuria and Katakwi where the NGO, Transcultural Psychosocial Organisation (TPO) was carrying out a psychosocial project to address HIV vulnerability in this war affected community. The sampling unit was a vulnerable person in the selected households. Only one vulnerable person was selected per household.

The formula for sample size calculation was [19]:

$$ n=\frac{Z^2pq}{d^2} $$

In the above equation, n is the sample size, z is the t-statistic for 95 % confidence interval, p the probability of the event, q = 1-p, and d level of precision desired. Using data from the Uganda Demographic Health Survey, 2006 for eastern Uganda [20], values for the components of the above formula were generated on eight vulnerability variables picked from the UDHS, 2006 report and used to calculate an implied sample size for each of the vulnerability variables. For example on the variable ‘proportion of population aged 15 to 19 years who cannot read at all’ the following component values were used z = 1.96, *p* = 0.224, q = 0.776, d = 0.05 to calculate an implied sample size of 267.1. The implied sample size values obtained for the eight vulnerability variables were then used to come up with a composite sample size number for this study, which was 268 per sub-county.

To realise the above sample size per sub-county, 15 villages per sub-county were randomly selected. In each village a comprehensive listing of vulnerable and non-vulnerable individuals by household was done with the help of village leaders. In each of the selected villages two tables of random numbers (one for vulnerable households, the other for non-vulnerable households) were generated and used to select households from which study respondent were recruited. In each of the selected household only one individual who met the vulnerability criteria/or did not meet this criteria (depending on whether this household was in the vulnerability or non-vulnerability arm of this study) was selected by simple random sampling. Eighteen vulnerable individuals and 6 non-vulnerable individuals (the comparison group) (one third of the vulnerable sample) were randomly selected from each village for interview (the proportion of one third for non-vulnerable individuals was arrived at to allow for statistical comparison on the vulnerability/ non-vulnerability category where required). In total, a sample of 1440 was calculated which was adjusted to 1584 (+10 % to take care of possible losses). A total of 1560 respondents were interviewed with 24 respondents repeatedly not found at home.

### Data collection

Non-medical interviewers who had previously participated in demographic surveillance data collection exercises with the Uganda Bureau of Statistics were recruited as research assistants and trained into basic concepts of psychological distress and into use of the different study modules.

### Measures

The data collection tool contained the following modules:

#### The dependent variable

The dependent variable in this study was *Intimate partner violence* (IPV) victimisation which was assessed by means of an 11-item questionnaire derived from the Intimate Partner Violence Assessment Questionnaire used by the American Congress of Obstetricians and Gynaecologists (2016) [21]. It was locally adapted by the inclusion of the following additional items: ‘being thrown out of one’s marital home by a spouse’, ‘having one’s children thrown out of the home’, ‘spouse taking up another partner’, ‘spouse giving away property to a lover without seeking the consent of one’s partner’. This instrument was asked to respondent who were married or had ever been married/or been in an intimate relationship, responses to the items being either yes or no. No formal validation of this questionnaire has ever been undertaken in the Ugandan context. The Cronbach’s alpha of this questionnaire was 0.857 in this study.

The following four derived variables were used in this analysis: ‘Any form of IPV’ (made up by having any of the 11 items of the questionnaire); ‘Sexual IPV’ (made up by having the item, ‘force you to have sex when you don’t want to?’); ‘Physical IPV’ (made up by having the item, ‘throw you down, push, hit, kick, slap, beat, or threaten you with a weapon?’); and ‘Psychological IPV’ (made up by having any of the following items: ‘threaten to hurt you?’, ‘threaten to hurt your children?’, ‘says it is your fault if he or she hits you, then promises it won’t happen again, but does it again?’, ‘put you down in public or keeps you from contacting family or friends?’, ‘insult you calling you ugly, says unpleasant things to you?, ‘did you partner ever threaten to chase you out of your matrimonial home?’, ‘did your partner ever chase you out of your matrimonial home?’, ‘got another partner’, and ‘did your spouse giving away property without your consent to a new lover?’).

The independent variables were

i) *Socio-demographics:* a) sub-county; b) sex; c) age in completed years; d) tribe; e) religion; f) marital status; and g) highest level of educational attainment.
ii) *Poverty index:* A poverty index was constructed from the 18 items from the definition of poverty of the Uganda Participatory Poverty Assessment Project [22]. For each item, a respondent was required to indicate whether: 0 = does not apply; 1 = mildly applies; 2 = moderately applies and 3 = significantly applies.
iii) *War torture experiences:* These items were derived from the commonly reported forms of war trauma in Africa [6, 14, 18] grouped under: a) *loss of loved ones*: (5 items)- including death as a result of war of the spouse, children, parents or other close relatives; b) *war related sexual torture:* (11 items)- including single episode rape, gang rape, homosexual rape, sexual comforting and abduction with sex; c) *physical torture:* (10 items)-including whether the respondent suffered beatings or being kicked, gunshot injury, burn injuries, being forced to carry heavy loads for long distances and severe tying of the hands behind the back.

#### Mental health problems

The study assessed the following:

iv) *Probable Major depressive disorder (PMDD)*: This was done using the 15-item Hopkins Symptom Checklist (HSCL-25) [23]. The items of the checklist are assessed using a Likert scale consisting of four items where: 1 = ‘not at all’; 2 = ‘a little’; 3 = 'quite a bit’ and 4 = ‘extremely’. A cut-off point of 31 has previously been calibrated by Kinyanda and colleagues (2009) as indicative of probable MDD [24] in the Ugandan situation. No formal validation of this instrument in the post-conflict situation of eastern Uganda was undertaken in this study. The Cronbach’s alpha of the Hopkins Symptom Checklist (HSCL-25) [23] in this study was 0.835.
v) *Probable Problem drinking (PPD) of alcohol:* ‘Probable problem drinking’ (PPD) for purposes of this study was defined as being equivalent to ‘alcoholism’ as defined by Ewing (1984) [25]. Ewing (1984) developed a four item questionnaire that asks about ‘the need to *C*ut down drinking’, ‘feeling *A*nnoyed by others’ criticism of their drinking’, ‘*G*uilt over drinking’, and ‘needing a drink as an *E*ye-opener in the morning’; thus the C.A.G.E. acronym. Validation of this questionnaire gave a cut off of two or more positive responses as being indicative of alcoholism [25]. This tool has subsequently been used successfully in two large epidemiological studies conducted in Africa [26, 27]. This tools was however not validated in the post-conflict situation of eastern Uganda. The Cronbach’s alpha of the C.A.G.E. in this study was 0.598.
vi) *Attempted suicide in the last 12-month*: This was assessed by means of the questions: ‘in the previous 12 months, have you attempted to take your life by ingesting poison, hanging, taking a drug overdose and drowning?’

The mental health modules used in the data collection tool was translated into Itesot (the local language spoken in the region) by forward and back translation by two teams of mental health professionals conversant with both English and the local language. When translating the mental health modules from English into Itesot the mental health team aimed to transmit into the local language the underlying mental health concept behind the constituent items of the module. After translation into the local language, both the English and local language versions of the data collection tool were compared at a consensus building meeting of the two translation teams. Where there was wide variations on individual items of the different modules between the English and local language version, the local version was revised according to the recommendations of the consensus building meeting. The whole process was supervised and overseen by the first author E.K. who is a trained psychiatrist.

### Ethical Issues

The study sought and obtained Ethical clearance from the Uganda National Council of Science and Technology [17]. Informed written consent was sought from study participants after adequate explanation of the study objectives and expected benefits to HIV prevention service development in the country. Those found to have significant scores on the various mental health assessment scales were offered a referral to the TPO supported clinics where a psychiatric nurse runs a mental health clinic in each of the sub-counties.

### Analysis

**Fig. 1:**
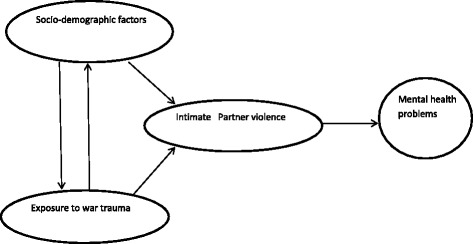
Conceptual framework guiding the Intimate Partner Violence analyses

Analysis was guided by a conceptual framework (Fig. 1) which considered the assessed variables as either risk factors of intimate partner violence (IPV) victimisation (socio-demographic factors and war trauma experiences) or as mental health consequences of IPV victimisation (‘probable problem drinking’, ‘attempted suicide in the 12 months’, and ‘probable major depressive disorder’) based on the hypotheses that war torture is a risk factor of IPV victimisation and that mental disorders are a consequence of IPV victimisation.

The primary outcome in this study was intimate-partner violence (IPV) victimisation, which was categorized as ‘any form of IPV’, ‘physical IPV’, ‘sexual IPV’ or ‘psychological IPV’. Multivariable logistic regression was used to assess factors associated with each outcome, weighted to allow for the sampling strategy. Factors associated with each outcome were analysed using weighted logistic regression, for males and females separately. Factors associated with the outcome in the univariate analysis (*p* < 0.20) were included in a multivariable model. Those factors independently associated with the outcome in the multivariable model (*p* < 0.10) were retained in the final model. Further analyses explored ‘probable problem drinking’ (PPD), ‘attempted suicide in the 12 months’, and ‘probable major depressive disorder’ (PMDD) as mental health consequences of IPV. Associations between each of these outcomes and IPV were adjusted for socio-economic factors shown to be associated with IPV in the earlier analyses.

## Results

### General Characteristics

Results of 1,560 respondents were collected and entered at data entry, however for this analysis only 1110 records were used (450 records had incomplete items and were not used). In this study males were 43.1 % while females were 56.9 %. The majority of the respondents (56 %) were aged between 18 to 44 years. Most respondents (89.5 %) had low educational attainment (no formal educational or less than eight years of formal schooling) with the main (81.3 %) economic activity being peasantry farming. The war torture experiences reported included: having a close relative killed (43.2 %), sexual torture (15.7 %), physical torture (48.3 %) and psychological torture (93.9 %). On mental health problems the following was reported: ‘probable major depressive disorder’ (PMDD) (41.6 %), ‘probable problem drinking’ (PPD) (16.9 %) and life-time attempted suicide (9.2 %).

A detailed description of the characteristics of the study respondents can be found in two previous publications from this study [15, 16].

### Prevalence of Intimate Partner Violence victimisation by gender

Table 1, the prevalence of ‘any form of IPV’ victimisation in this study was 43.7 % [95 % CI, 40.1–47.4 %], there was no statistically significant difference on prevalence of ‘any form of IPV’ by gender (females 44.9 % [95 % CI, 39.8–50.0 %], males 41.8 % [95 % CI, 37.2–46.7 %]; χ^2^ = 0.943, *p* = 0.331). There was no statistically significant difference on prevalence of the different forms of IPV by gender: ‘sexual IPV’ (females 7.8 % [95 % CI, 5.9–10.2 %], males 5.4 % [95 % CI, 3.6–7.9 %]; χ^2^ = 2.534, *p* = 0.111); ‘physical IPV’(females 22.9 % [95 % CI, 19.5–26.7 %], males 21.5 % [95 % CI, 17.8–25.7 %]; χ^2^ = 0.345, *p* = 0.557) and ‘psychological IPV’ (females 44.2 % [95 % CI, 39.1–49.3 %], males 39.3 % [95 % CI, 34.6–44.1 %]; χ^2^ = 2.323, *p* = 0.128).

**Table 1:** Prevalence of the different forms of intimate partner violence by gender

	Total sample (*N* = 1110)	Female (*n* = 694)	Male (*n* = 416)	Chi-square	*P* value
	Prevalence (95 % CI)	Prevalence (95 % CI)	Prevalence (95 % CI)		
Any IPV	43.7 % (40.1–47.4 %)	44.9 % (39.8–50.0 %)	41.8 % (37.2–46.7 %)	0.943	0.331
Sexual IPV	6.9 % (5.5–8.6 %)	7.8 % (5.9–10.2 %)	5.4 % (3.6–7.9 %)	2.534	0.111
Physical IPV	22.4 % (19.8–25.2 %)	22.9 % (19.5–26.7 %)	21.5 % (17.8–25.7 %)	0.345	0.557
Psychological IPV	42.4 % (38.8–46.0 %)	44.2 % (39.1–49.3 %)	39.3 % (34.6–44.1 %)	2.323	0.128

### Risk factors of IPV

Table 2, shows the univariate analyses between socio-demographic factors and war torture experiences and the different forms of IPV victimisation by gender. The factors significantly associated with the different forms of IPV victimisation among females were: ‘physical IPV’ sub-county (least in the sub-county of Acowa), highest educational attainment (more among those with a primary level education than those with no formal education), use of alcohol (more among those reporting alcohol use than those who did not use alcohol); ‘sexual IPV’ use of alcohol (more among those reporting alcohol use than those who did not use alcohol); ‘psychological IPV’ use of alcohol (more among those reporting alcohol use than those who did not use alcohol). Among males the only socio-demographic factor significantly associated with both ‘physical IPV’ and ‘psychological IPV’ was increasing levels of poverty (we did not undertake a risk factor analysis for ‘sexual IPV’ among males because the numbers of males reporting ‘sexual IPV’ was small).

**Table 2:** The association between socio-demographics and war torture experiences with IPV

	Males		Females		
	Physical IPV	Psychological IPV	Physical IPV	Psychological IPV	Sexual IPV
	Univariate OR (95 % CI)	Univariable OR (95 % CI)	Univariate OR (95 % CI)	Univariable OR (95 % CI)	Univariate OR (95 % CI)
	90/416 (21.5 %)	164/416 (39.3 %)	159/694 (22.9 %)	307/694 (44.2 %)	54/694 (7.8 %)
Sub-county	*P* = 0.40	*P* = 0.75	*P* = 0.004	*P* = 0.07	*P* = 0.68
Acowa	1	1	1	1	1
Magoro	1.60 (0.84-3.03)	0.98 (0.56-1.72)	2.28 (1.20-4.33)	2.02 (1.11-3.68)	0.66 (0.28-1.55)
Ngariam	0.96 (0.49-1.87)	0.89 (0.52-1.51)	3.16 (1.67-5.96)	2.19 (1.20-4.01)	0.76 (0.32-1.78)
Usuk	1.19 (0.61-2.33)	1.21 (0.69-2.11)	2.78 (1.45-5.30)	2.01 (1.10-3.68)	1.02 (0.46-2.29)
Socio-demographics					
Age (years)	*P* = 0.21	*P* = 0.75	*P* = 0.40	*P* = 0.55	*p* = 0.50
15-24	1	1	1	1	1
25-34	0.90 (0.40-2.00)	0.74 (0.36-1.53)	1.62 (0.82-3.21)	1.05 (0.60-1.85)	0.94 (0.33-2.67)
35-44	0.96 (0.43-2.15)	0.70 (0.34-1.46)	1.14 (0.52-2.48)	0.78 (0.37-1.67)	1.34 (0.50-3.59)
45-54	0.42 (0.16-1.14)	0.76 (0.34-1.69)	1.01 (0.49-2.09)	1.00 (0.56-1.79)	0.39 (0.10-1.42)
55-64	0.65 (0.23-1.81)	0.54 (0.22-1.34)	1.27 (0.59-2.75)	0.63 (0.33-1.18)	1.19 (0.39-3.61)
65+	0.43 (0.16-1.14)	0.56 (0.25-1.26)	1.73 (0.85-3.55)	0.81 (0.44-1.48)	0.94 (0.32-2.78)
Marital status	*P* = 0.09	*P* = 0.14	*P* = 0.69	*P* = 0.12	*P* = 0.54
Married	1	1	1	1	1
Divorced/widowed	0.48 (0.21-1.12)	0.62 (0.33-1.18)	0.92 (0.60-1.40)	0.71 (0.47-1.09)	0.83 (0.45-1.52)
Education level	*P* = 0.10	*P* = 0.14	*P* = 0.03	*P* = 0.22	*P* = 0.86
No education	1	1	1	1	1
Primary only	1.77(1.04-3.03)	1.44 (0.93-2.23)	1.50 (1.00-2.24)	1.37 (0.93-2.02)	0.95 (0.52-1.74)
Secondary or higher	1.68 (0.79-3.54)	1.67 (0.89-3.14)	0.20 (0.03-1.53)	0.84 (0.28-2.50)	
Religion	*P* = 0.58	*P* = 0.98	*P* = 0.24	*P* = 0.29	*P* = 0.74
Catholic	1	1	1	1	1
Protestant	1.04 (0.63-1.73)	1.01 (0.66-1.56)	1.22 (0.80-1.87)	1.26 (0.84-1.87)	1.28 (0.68-2.41)
Other	0.34 (0.04-2.76)	0.89 (0.25-3.33)	2.07 (0.82-5.22)	1.82 (0.74-4.44)	1.14 (0.25-5.12)
Poverty index	*P* = 0.0006	*P* = 0.005	*P* = 0.24	*P* = 0.86	*P* = 0.28
Low (≤23)	1	1	1	1	1
Medium (24–30)	1.94 (0.96-3.91)	1.70 (0.99-2.90)	1.46 (0.91-2.33)	1.08 (0.73-1.59)	0.59 (0.27-1.26)
High (> = 30)	3.57 (1.83-6.98)	2.44 (1.43-4.15)	1.45 (0.83-2.52)	1.16 (0.66-2.03)	1.03 (0.50-2.09)
Alcohol use	*P* = 0.15	*P* = 0.09	*P* = 0.003	*P* = 0.006	*P* = 0.02
Non drinker	1	1	1	1	1
Drinker	1.41 (0.88-2.27)	1.42 (0.95-2.12)	1.82 (1.22-2.72)	1.70 (1.16-2.50)	2.06 (1.14-3.71)
War-related					
torture experiences					
Close relative killed^1^	*P* = 0.29	*P* = 0.97	*P* = 0.26	*P* = 0.56	*P* = 0.06
No	1	1	1	1	1
Yes	0.77 (0.48-1.24)	1.00 (0.67-1.50)	1.27 (0.84-1.93)	0.89 (0.59-1.34)	1.81 (0.97-3.36)
Sexual torture	*P* = 0.16	*P* = 0.006	*P* = 0.02	*P* = 0.02	*P* = 0.12
No	1	1	1	1	1
Yes	1.56 (0.84-2.92)	2.20 (1.25-3.88)	1.70 (1.08-2.68)	1.71 (1.11-2.64)	1.70 (0.88-3.29)
Physical torture	*P* = 0.68	*P* = 0.009	*P* = 0.002	*P* = 0.009	*P* = 0.007
No	1	1	1	1	1
Yes	1.10 (0.69-1.77)	1.72 (1.15-2.57)	1.98 (1.30-3.03)	1.73 (1.15-2.60)	2.45 (1.28-4.67)

Note

^1^Includes parent, child, spouse or sibling (not other relative as this was too common)

On the association between war torture experiences and IPV victimisation, among females: ‘physical IPV’ was associated with both sexual torture and physical torture; ‘sexual IPV’ with sexual torture; and ‘psychological IPV’ with both sexual torture and physical torture. Among males, only ‘psychological IPV’ was associated with both sexual torture and physical torture.

### Multivariate analysis of risk factors of IPV victimisation

Table 3 shows the risk factors independently significantly associated with the different forms of IPV victimisation by gender. Among females the risk factors independently associated with the different types of IPV victimisation were: ‘physical IPV’ sub-county, highest educational attainment, alcohol use and physical torture; ‘psychological IPV’ sub-county, marital status (less among the divorced/separated compared with the married), alcohol use and physical torture experiences; ‘sexual IPV’ alcohol use and physical torture experience. Among males the factors independently associated with the different forms of IPV victimisation were: ‘physical IPV’ poverty; ‘psychological IPV’ poverty, sexual torture and physical torture.

**Table 3:** Factors independently significantly associated with the different IPV

	Males		Females		
	Physical IPV	Psychological IPV	Physical IPV	Psychological IPV	Sexual IPV
	Multivariable OR^1^ (95 % CI)	Multivariable OR^1^ (95 % CI)	Multivariable OR^1^ (95 % CI)	Multivariable OR^1^ (95 % CI)	Multivariable OR^1^ (95 % CI)
Sub-county	-	-	*P* = 0.002	*P* = 0.02	-
Acowa			1	1	
Magoro			2.32 (1.27-4.24)	2.05 (1.22-3.46)	
Ngariam			3.16 (1.75-5.70)	2.19 (1.29-3.70)	
Usuk			2.63 (1.44-4.81)	1.88 (1.12-3.15)	
Education level	-	-	*P* = 0.02		-
No education			1		
Primary only			1.60 (1.08-2.37)		
Secondary 1+			0.25 (0.03-1.91)		
Marital status	-	-		*P* = 0.03	-
Married				1	
Divorced/widowed				0.67 (0.47-0.97)	
Poverty index	*P* = 0.0006	*P* = 0.02	-		-
Low (≤23)	1	1			
Medium (24–30)	1.94 (0.96-3.91)	1.87 (1.07-3.26)			
High (> = 30)	3.67 (1.83-6.98)	2.17 (1.26-3.74)			
Sexual torture		*P* = 0.04		*P* = 0.10	-
No		1		1	
Yes		1.82 (1.02-3.23)		1.44 (0.94-2.22)	
Close relative killed^1^	-	-	-		*P* = 0.07
No					1
Yes					1.74 (0.95-3.20)
Physical torture	-	*P* = 0.02	*P* = 0.001	*P* = 0.001	*P* = 0.01
No		1	1	1	1
Yes		1.64 (1.07-2.49)	2.03 (1.36-3.03)	1.74 (1.21-2.50)	2.34 (1.22-4.49)
Alcohol consumption -		*P* = 0.08	*P* = 0.006	*P* = 0.01	*P* = 0.07
Non drinker		1	1	1	1
Drinker		1.74 (1.18-2.59)	1.74 (1.18-2.59)	1.58 (1.11-2.27)	1.95 (1.08-3.53)

Note ^1^Includes parent, child, spouse or sibling (not other relative as this was too common)

### Association between IPV victimisation and mental health consequences

Table 4 shows the association between the different forms of IPV victimisation and mental health problems. Among females the mental health problems that were significantly associated with different forms of IPV victimisation were: ‘physical IPV’ attempted suicide in the last 12-months; ‘psychological IPV’ probable problem drinking; ‘sexual IPV’ probable problem drinking, attempted suicide in the last 12 months and probable major depressive disorder. Among males only probable problem drinking was significantly associated with ‘psychological IPV’.

**Table 4:** Association between physical, psychological and sexual IPV and mental health consequences

Psychiatric consequences	Males		Females		
	Physical IPV	Psychological IPV	Physical IPV	Psychological IPV	Sexual IPV
	Adjusted OR^1^ (95 % CI)	Adjusted OR^1^ (95 % CI)	Adjusted OR^1^ (95 % CI)	Adjusted OR^1^ (95 % CI)	Adjusted OR^1^ (95 % CI)
Probable problem alcohol drinking	*P* = 0.05	*P* = 0.03	*P* = 0.08	*P* = 0.001	*P* = 0.02
Non problem drinker	1	1	1	1	1
Problem drinker	1.69 (1.00-2.85)	1.68 (1.05-2.67)	1.55 (0.95-2.52)	2.12 (1.37-3.31)	2.29 (1.17-4.44)
Attempted suicide in past 12 months	*P* = 0.22	*P* = 0.46	*P* = 0.01	*P* = 0.06	*P* = 0.005
No	1	1	1	1	1
Yes	2.20 (0.55-8.78)	1.71 (0.41-7.22	3.02 (1.29-7.07)	2.14 (0.96-4.78)	4.20 (1.54-11.46)
Probable major depressive disorder	*P* = 0.70	*P* = 0.83	*P* = 0.08	*P* = 0.86	*P* = 0.03
No	1	1	1	1	1
Yes	0.90 (0.54-1.51	0.95 (0.61-1.48)	1.41 (0.96-2.07)	0.97 (0.68-1.39)	2.15 (1.09-4.23)

Note: ^1^Adjusted for sub-county, marital status, education and poverty category (SES factors associated with IPV in Table 2)

## Discussion

This is one of the first published studies that has investigated in a post-conflict situation in sub-Saharan Africa the relationship between war torture experiences, IPV victimisation and mental health problems. The principal findings of this study is that intimate partner violence (IPV) victimisation is highly prevalent in post-conflict eastern Uganda afflicting nearly half of all respondents 18 years above, there was however no statistically significant difference in the rates of IPV between the two gender. Secondly, in line with the set hypothesis, in both gender, war torture was a risk factor of IPV victimisation and IPV victimisation was associated with a number of mental health problems.

A considerable number (43.7 %) of respondents in this study reported a life-time experience of any form of IPV victimisation (physical, sexual or psychological). Comparing the results of this study for any form of IPV (female 44.9 %, male 43.7 %) with those from the Uganda Demographic Health Survey (UDHS), 2011 for eastern region (region where this study was undertaken) (females 71.3 %, males 43.6 %) showed similar rates among males but lower female rates in this study [7]. The rates of IPV reported in this study are similar to those reported in other conflict affected communities in sub-Saharan Africa. Hossain and colleagues (2015) among post-conflict rural communities in Ivory Coast reported lifetime physical and/or sexual IPV among ever-partnered females of 49.8 % and lifetime physical IPV among males of 19.8 % [4]. In the Democratic Republic of the Congo among adult females who were married/cohabiting, Tlapek (2014) reported a rate for any form of IPV of 68.2 % [28]. Kinyanda and colleagues (2009) in four conflict affected counties of Liberia reported a lifetime rate for any form of IPV of 35.6 % [6]. The above rates of IPV from conflict affected communities in sub-Saharan Africa are much higher than those reported from non-conflict affected sub-Saharan African countries, Gass and colleagues (2011) in South Africa among married/cohabiting adults reported a rate for physical IPV of 29.3 % among females and 20.9 % among males, while Andersson and colleagues (2007) in a eight country study among adults in southern Africa reported a rate of physical IPV of 18 % among females and 14 % among males [29, 30].

In this study, there was no statistically significant difference in the rates of IPV between the two gender. Similar to this study, a negligible gender difference in rates of IPV has been reported by Andersson and colleagues (2007) in southern Africa where in an eight country study, they reported a negligible gender gap in IPV rates in five of the eight study countries [30]. In this study gender symmetry in rates of IPV was observed on all three types of IPV. Reasons for gender symmetry observed in some African communities but not others are not immediately clear but may include cultural beliefs and practices that may be specific to given communities. Although the rates of IPV victimisation in this study were found to be similar in both gender, there may be gender differences in the meaning of IPV victimisation and in the severity of IPV victimisation [29]. There is however need for more research in this area to better understand gender symmetry of IPV victimisation in the sub-Saharan African setting.

In this study the socio-demographic factors that were significantly associated with IPV victimisation among females were sub-county (representing ecological factors that may include cultural beliefs and practices that may be specific to given community), use of alcohol and higher educational attainment (although there was no definite trend on educational attainment). Among males it was only poverty that was significantly associated with IPV victimisation. It is not immediately clear why the female residents of the sub-county of Acowa reported significantly less physical IPV (a relationship that retained significance at multivariate analysis) compared to the other sub-counties in the same region. Jewkes (2002) reports that sometimes the risk for IPV varies between otherwise similar settings within countries and offers the following possible reasons to explain this observation: differences between communities on willingness to disclose violent experiences at interview, cultural differences in the status of women, and cultural differences in the acceptability of interpersonal violence, he however calls for more research to better understand this phenomenon [31]. In this study, alcohol use among females was a significant risk factor for all forms of IPV victimisation a finding which has been reported by others [29, 31, 32]. Among males in this study increasing levels of poverty were significantly associated with IPV victimisation a finding which was observed in the UDHS, 2011 and has also been reported by other investigators [7, 29, 31].

Previous experience of war torture was associated with IPV victimisation in both gender in this study. In both gender the physical forms of torture (sexual and physical torture) but not the psychological forms of torture (having a close relative killed) were associated with the different forms of IPV. We did not come across literature that described the association between previous war torture experiences and IPV victimisation. The nearest literature to this was that which described the association between experiencing childhood trauma and witnessing parental violence and later IPV victimisation [29]. Shamu and colleagues (2011) in a systematic review of African studies on IPV in pregnant women observed that a history of violence was significantly associated with IPV victimisation in pregnancy [32]. To explain the association between previous trauma and IPV, Thaler (2012) has suggested that exposure to violence normalises it and may then lead either to future perpetration or victimisation [33]. Another possible mechanism that would explain the link between previous war torture and future IPV victimisation could be through war torture induced mental disorders such as post traumatic stress disorder (PTSD). Krause and colleagues (2006) in a prospective study of women exposed to IPV observed that the numbing symptoms of PTSD at baseline increased the risk for future IPV victimisation [34]. There is however need for more research including longitudinal studies to better understand the link between war torture and IPV victimisation.

In this study the different forms of IPV victimisation were significantly associated with mental health problems in both gender, however IPV victimisation in females was associated with a broader range of mental health problems. The mental health problems associated with IPV victimisation among females were problem alcohol drinking, attempted suicide and depression while among males it was only problem alcohol drinking. Physical, sexual and psychological IPV were all significantly associated with mental health problems. Previous studies from the industrialised countries have reported the association of IPV with depressive symptoms, alcohol and substance use/abuse and suicidal tendencies [35–37]. Gupta and colleagues (2014) from war affected rural Ivory Coast reported a robust positive association between IPV and probable PTSD with the more severe forms of IPV such sexual violence and choking showing heighted odds of association with probable PTSD [10].

## Conclusion

In conclusion, in line with the set hypothesis of this study, war torture was significantly associated with IPV victimisation and the mental health problems of depression, problem drinking and suicide attempted were significantly associated with various forms of IPV victimisation. However given that the relationship between IPV victimisation and some of the mental health problems such alcohol abuse is bidirectional [37] and that war torture could directly lead to some of the investigated mental health problems [6–8, 18], there is need for longitudinal studies to elucidate the actual direction of causality between these investigated factors.

Additional limitations of this study include that: a formal power calculation at sample size determination was not done for this study which could have affected the results of some of the multivariate analyses; the modified Intimate Partner Violence Assessment Questionnaire [20] that was used to assess for IPV in this study although locally adapted has never been formally validated in the post-conflict situation of eastern Uganda, it however had a good reliability index (Cronbach’s alpha =0.857); the tools that were used to assess for mental health problems in this study although translated into the local language were not formally validated in the study communities, they however had good reliability indices (the measure for probable major depressive disorder, the Hopkins Symptom Checklist [23] had a Cronbach’s alpha of 0.835, while the measure for probable problem drinking the C.A.G.E [25] had a Cronbach’s alpha of 0.598.

As recommendations, the mental health rehabilitation of conflict and post-conflict communities should consider the co-occurrence of the psychosocial problems of war torture and IPV victimisation and should therefore incorporate screening for the later in all psychosocial assessments of war torture survivors. Secondly, in conflict and post-conflict settings in Africa, IPV victims should be considered as a risk category for mental disorders.

## Abbreviations

IPV: intimate partner violence
PTSD: post traumatic stress disorder
LRA: Lord’s Resistance Army
UPA: Uganda People’s Army
TPO: Transcultural Psychosocial Organisation
